# Crystal structure of bis­[μ-bis­(di­phenyl­phosphan­yl)methane-κ^2^ *P*:*P*′]-μ-chlorido-chlorido-1κ*Cl*-(1-phenyl­thio­urea-2κ*S*)disilver aceto­nitrile hemisolvate

**DOI:** 10.1107/S2056989015008981

**Published:** 2015-05-23

**Authors:** Arunpatcha Nimthong-Roldán, Janejira Ratthiwal, Yupa Wattanakanjana

**Affiliations:** a Department of Chemistry, Youngstown State University, 1 University Plaza, 44555 Youngstown, OH, USA; b Department of Chemistry, Faculty of Science, Prince of Songkla University, Hat Yai, Songkhla 90112, Thailand

**Keywords:** crystal structure, silver complex, *N*,*N*′-phenyl­thio­urea, hydrogen bonding

## Abstract

In the dinuclear title complex, [Ag_2_Cl_2_(C_7_H_8_N_2_S)(C_25_H_22_P_2_)_2_]·0.5CH_3_CN, each Ag^I^ ion displays a distorted tetra­hedral coordination geometry with two P atoms from two bis­(di­phenyl­phosphan­yl)methane (dppm) ligands, one bridging chloride ion, one terminal chloride ion and one terminal S atom from the *N*,*N*′-phenyl­thio­urea (ptu) ligand. The dppm ligands and the bridging chloride ion force the two Ag atoms into close proximity, with a short Ag⋯Ag separation of 3.2064 (2) Å. In the crystal, complex mol­ecules are linked by N—H⋯Cl hydrogen bonds forming a dimer. The dimers are linked *via* weak C— H⋯Cl hydrogen bonds forming a two-dimensional supra­molecular architecture in the *yz* plane. In addition, an intra­molecular N—H⋯Cl hydrogen bond is observed.

## Related literature   

For the studies of silver(I) complexes containing phosphine and sulfur co-donor ligands, see: Zhang *et al.* (2003 ▸); Wattanakanjana *et al.* (2013 ▸). For their various applications such as anti­microbial activities, see: Isab *et al.* (2010 ▸); Nawaz *et al.* (2011 ▸).
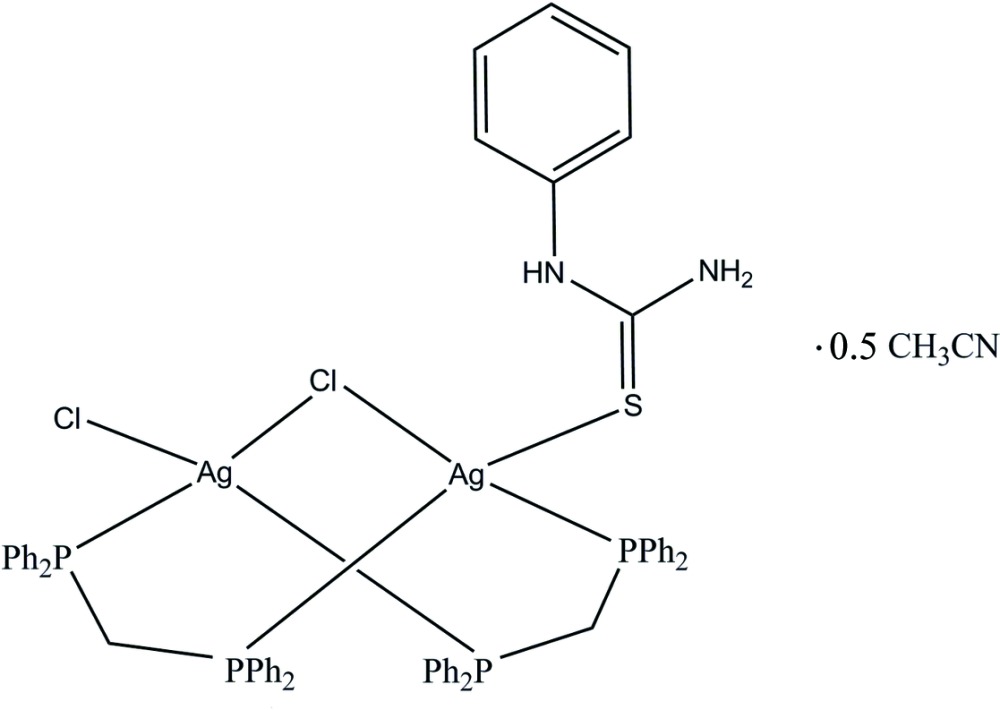


## Experimental   

### Crystal data  

[Ag_2_Cl_2_(C_7_H_8_N_2_S)(C_25_H_22_P_2_)_2_]·0.5C_2_H_3_N
*M* *_r_* = 2456.22
Monoclinic, 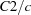

*a* = 30.6130 (12) Å
*b* = 16.5078 (4) Å
*c* = 21.7975 (6) Å
β = 97.129 (2)°
*V* = 10930.3 (6) Å^3^
*Z* = 4
Mo *K*α radiation
μ = 1.01 mm^−1^
*T* = 100 K
0.38 × 0.27 × 0.05 mm

### Data collection  

Bruker AXS D8 Quest CMOS diffractometer
Absorption correction: multi-scan (*SADABS*; Bruker, 2014 ▸) *T*_min_ = 0.564, *T*_max_ = 0.746
43370 measured reflections
13309 independent reflections
11256 reflections with *I* > 2σ(*I*)
*R*_int_ = 0.038

### Refinement  

*R*[*F* ^2^ > 2σ(*F* ^2^)] = 0.031
*wR*(*F* ^2^) = 0.076
*S* = 1.09
13309 reflections
628 parameters
H-atom parameters constrained
Δρ_max_ = 1.11 e Å^−3^
Δρ_min_ = −0.82 e Å^−3^

### 

Data collection: *APEX2* (Bruker, 2014 ▸); cell refinement: *SAINT* (Bruker, 2014 ▸); data reduction: *SAINT*; program(s) used to solve structure: *SHELXS97* (Sheldrick, 2008 ▸); program(s) used to refine structure: *SHELXL2013* (Sheldrick, 2015 ▸) and *SHELXLE* (Hübschle *et al.*, 2011 ▸); molecular graphics: *Mercury* (Macrae *et al.*, 2008 ▸); software used to prepare material for publication: *SHELXL2013*.

## Supplementary Material

Crystal structure: contains datablock(s) I, New_Global_Publ_Block. DOI: 10.1107/S2056989015008981/pj2019sup1.cif

Structure factors: contains datablock(s) I. DOI: 10.1107/S2056989015008981/pj2019Isup2.hkl

**Fig. 1.**
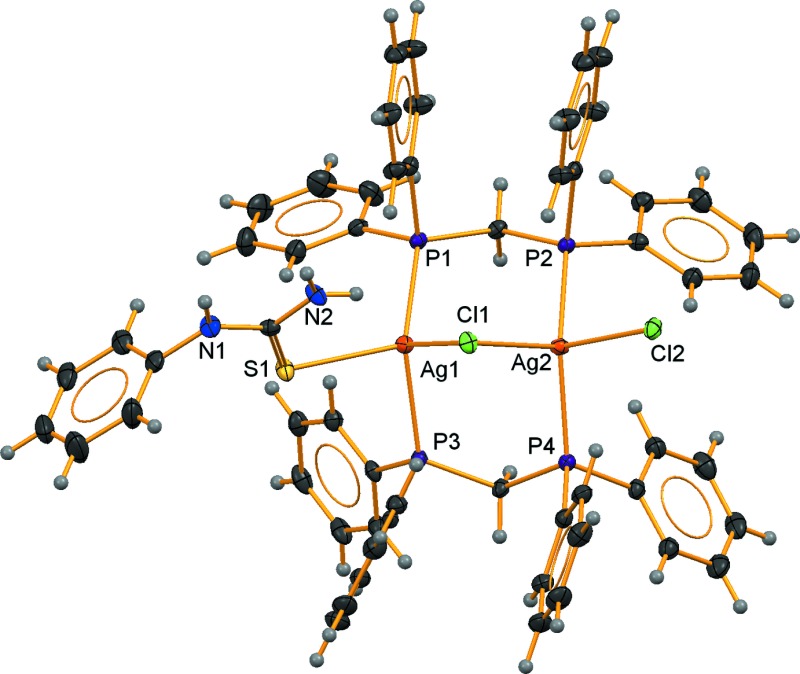
The molecular structure with displacement ellipsoids drawn at the 50% probability level. The acetonitrile is omitted for clarity.

. DOI: 10.1107/S2056989015008981/pj2019fig1.tif
The mol­ecular structure with displacement ellipsoids drawn at the 50% probability level. The aceto­nitrile is omitted for clarity.

**Fig. 2.**
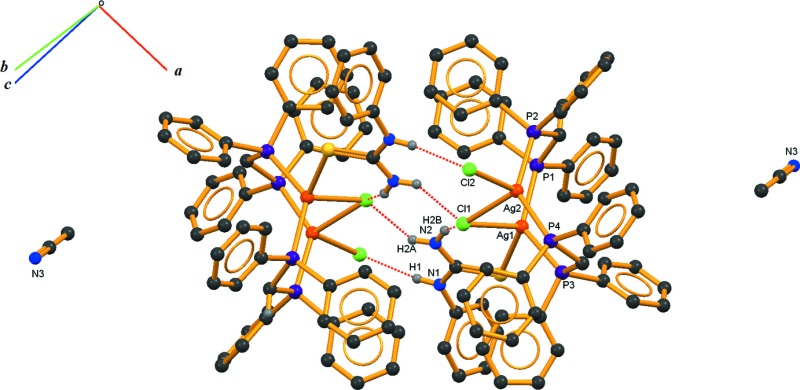
Part of the crystal structure showing intra-intermolecular N—H···Cl hydrogen bonds forming a dimers as dashed lines.

. DOI: 10.1107/S2056989015008981/pj2019fig2.tif
Part of the crystal structure showing intra-inter­molecular N—H⋯Cl hydrogen bonds forming a dimers as dashed lines.

**Fig. 3.**
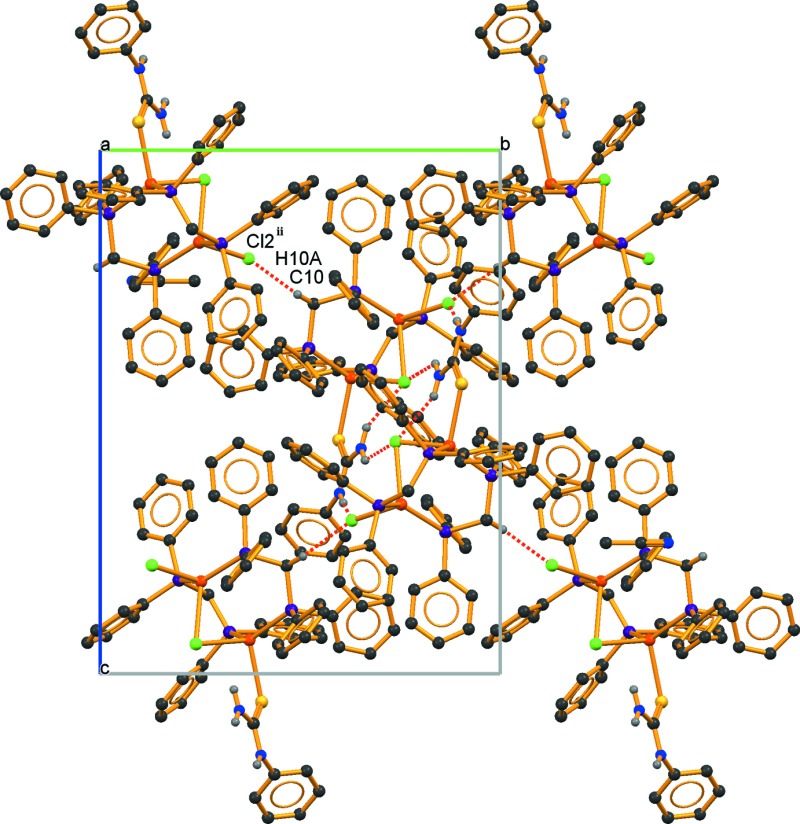
Part of the crystal structure showing intermolecular C—H···Cl hydrogen bonds as dashed lines, forming a two-dimensional network parallel to (100).

. DOI: 10.1107/S2056989015008981/pj2019fig3.tif
Part of the crystal structure showing inter­molecular C—H⋯Cl hydrogen bonds as dashed lines, forming a two-dimensional network parallel to (100).

CCDC reference: 1064097

Additional supporting information:  crystallographic information; 3D view; checkCIF report

## Figures and Tables

**Table 1:** Hydrogen-bond geometry (, )

*D*H*A*	*D*H	H*A*	*D* *A*	*D*H*A*
N1H1Cl2^i^	0.88	2.32	3.1910(19)	172
N2H2*A*Cl1^i^	0.88	2.56	3.1618(19)	126
N2H2*A*Cl2^i^	0.88	2.84	3.595(2)	144
N2H2*B*Cl1	0.88	2.52	3.328(2)	152

Symmetry code: (i) 
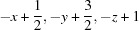
.

## supplementary crystallographic information

### S1. Experimental

Bis(di­phenyl­phosphanyl)methane, dppm, (0.1 g, 0.26 mmol) was dissolved in 30 ml of aceto­nitrile at 343 K and then silver(I) chloride, AgCl, (0.04 g, 0.28 mmol) was added. The mixture was stirred for 4 hr and then *N*,*N*′-phenyl­thio­urea, ptu, (0.04 g, 0.26 mmol) was added and the new reaction mixture was heated under reflux for 6 hr during which the precipitate gradually disappeared. The resulting clear solution was filtered and left to evaporate at room temperature. The crystalline complex, which deposited upon standing for several days, was filtered off and dried in vacuo.

### S1.1. Refinement

H atoms bonded to C and N atoms were included in calculated positions and were refined with a riding model using distances of 0.95 Å (aryl H), and *U_iso_*(H) = 1.2*U_eq_*(C); 0.99 Å (CH_2_) and *U_iso_*(H) = 1.5*U_eq_*(C); 0.88 Å (NH), and *U_iso_*(H) = 1.2*U_eq_*(N).

### S2. Results and discussion

Silver(I) complexes containing phosphine and sulfur co-donor ligands have been studied in recent years (Zhang *et al.*, 2003; Wattanakanjana *et al.*, 2013) because of their various applications such as anti­microbial activities (Isab *et al.*, 2010; Nawaz *et al.*, 2011). Herein, the crystal structure of a dinuclear silver(I) chloride complex containing both dppm and ptu is described.

The title complex is dinuclear in which the Ag^I^ ions adopt distorted tetra­hedral geometries. Bis(di­phenyl­phosphanyl)methane (dppm) affords a dinuclear metal complex in the µ-dppm form. There are two kinds of coordination environment around the Ag^I^ centres. In one of the Ag centre, the silver atom, Ag1, is tetra­hedrally coordinated to two P atoms of two dppm ligands, one bridging chloride ion and a terminal S atom of *N*,*N*'-phenyl­thio­urea (ptu) ligand. In the second centre, the silver atom, Ag2, is bound to two P atoms of two dppm ligands and two chloride ions form the bridging and a terminal Cl atom in a tetra­hedral environment as shown in Fig. 1. The dppm ligands and the bridging chloride ion force the two Ag atoms into close proximity with a short Ag···Ag separation of 3.2064 (2) Å. In the crystal, N1—H1···Cl2^i^ and N2—H2*A*···Cl1^i^ hydrogen bonds are linked molecules into dimers (Fig. 2 and Table 1). The dimers are connected *via* a weak C10— H10*A*···Cl2^ii^ hydrogen bonds [C10(*sp*^3^)—H10*A*···Cl2^ii^, with H10*A*···Cl2^ii^ = 2.6379 (6) Å, C10(*sp*^3^)···Cl2^ii^, = 3.581 (2) Å and C10(*sp*^3^)—H10*A*···Cl2^ii^, = 159.20 (14)°, symmetry code: (ii) 1/2 - x,-1/2 + y, 1/2 - z], leading to the formation of a two-dimensional network parallel to (100), Fig. 3. In addition, an intra­molecular N2—H2B···Cl1 hydrogen bond is observed.

### Crystal data

**Table:**

[Ag_2_Cl_2_(C_7_H_8_N_2_S)(C_25_H_22_P_2_)_2_]·0.5C_2_H_3_N	*F*(000) = 4984
*M**_r_* = 2456.22	*D*_x_ = 1.493 Mg m^−^^3^
Monoclinic, *C*2/*c*	Mo *K*α radiation, λ = 0.71073 Å
*a* = 30.6130 (12) Å	Cell parameters from 9809 reflections
*b* = 16.5078 (4) Å	θ = 2.3–28.3°
*c* = 21.7975 (6) Å	µ = 1.01 mm^−^^1^
β = 97.129 (2)°	*T* = 100 K
*V* = 10930.3 (6) Å^3^	Plate, colourless
*Z* = 4	0.38 × 0.27 × 0.05 mm

### Data collection

**Table:**

Bruker AXS D8 Quest CMOS diffractometer	13309 independent reflections
Radiation source: I-mu-S microsource X-ray tube	11256 reflections with *I* > 2σ(*I*)
Laterally graded multilayer (Goebel) mirror monochromator	*R*_int_ = 0.038
ω and phi scans	θ_max_ = 28.3°, θ_min_ = 2.3°
Absorption correction: multi-scan (*SADABS*; Bruker, 2014)	*h* = −40→37
*T*_min_ = 0.564, *T*_max_ = 0.746	*k* = −22→22
43370 measured reflections	*l* = −29→27

### Refinement

**Table:**

Refinement on *F*^2^	Primary atom site location: structure-invariant direct methods
Least-squares matrix: full	Secondary atom site location: difference Fourier map
*R*[*F*^2^ > 2σ(*F*^2^)] = 0.031	Hydrogen site location: mixed
*wR*(*F*^2^) = 0.076	H-atom parameters constrained
*S* = 1.09	*w* = 1/[σ^2^(*F*_o_^2^) + (0.0258*P*)^2^ + 25.3039*P*] where *P* = (*F*_o_^2^ + 2*F*_c_^2^)/3
13309 reflections	(Δ/σ)_max_ = 0.003
628 parameters	Δρ_max_ = 1.11 e Å^−^^3^
0 restraints	Δρ_min_ = −0.82 e Å^−^^3^

### Special details

**Table:**

Geometry. All e.s.d.'s (except the e.s.d. in the dihedral angle between two l.s. planes) are estimated using the full covariance matrix. The cell e.s.d.'s are taken into account individually in the estimation of e.s.d.'s in distances, angles and torsion angles; correlations between e.s.d.'s in cell parameters are only used when they are defined by crystal symmetry. An approximate (isotropic) treatment of cell e.s.d.'s is used for estimating e.s.d.'s involving l.s. planes.

### Fractional atomic coordinates and isotropic or equivalent isotropic displacement parameters (Å^2^)

**Table:**

	*x*	*y*	*z*	*U*_iso_*/*U*_eq_	Occ. (<1)
Ag1	0.34726 (2)	0.62699 (2)	0.43524 (2)	0.01319 (4)	
Ag2	0.31458 (2)	0.74785 (2)	0.32376 (2)	0.01379 (5)	
Cl1	0.30072 (2)	0.76058 (3)	0.44301 (3)	0.01595 (11)	
Cl2	0.26067 (2)	0.87057 (3)	0.29281 (3)	0.01781 (11)	
S1	0.37141 (2)	0.59639 (4)	0.55368 (3)	0.01809 (12)	
P1	0.30220 (2)	0.52187 (3)	0.37837 (3)	0.01281 (11)	
P2	0.27329 (2)	0.63632 (3)	0.27086 (3)	0.01257 (11)	
P3	0.42265 (2)	0.67660 (3)	0.42066 (3)	0.01213 (11)	
P4	0.38881 (2)	0.80300 (3)	0.32099 (3)	0.01273 (11)	
N1	0.33087 (6)	0.59822 (13)	0.65532 (9)	0.0194 (4)	
H1	0.3073	0.6076	0.6735	0.023*	
N2	0.29192 (6)	0.65224 (13)	0.56963 (9)	0.0184 (4)	
H2A	0.2706	0.6621	0.5922	0.022*	
H2B	0.2888	0.6657	0.5303	0.022*	
C1	0.32886 (7)	0.61682 (14)	0.59508 (11)	0.0147 (4)	
C2	0.36829 (8)	0.56437 (16)	0.69221 (11)	0.0217 (5)	
C3	0.37998 (8)	0.48414 (17)	0.68583 (12)	0.0239 (5)	
H3	0.3636	0.4507	0.6558	0.029*	
C4	0.41607 (9)	0.45264 (19)	0.72405 (13)	0.0291 (6)	
H4	0.4244	0.3977	0.7197	0.035*	
C5	0.43962 (10)	0.5012 (2)	0.76824 (15)	0.0394 (8)	
H5	0.4643	0.4798	0.7939	0.047*	
C6	0.42720 (12)	0.5803 (2)	0.77492 (17)	0.0502 (9)	
H6	0.4430	0.6133	0.8058	0.060*	
C7	0.39154 (11)	0.61243 (19)	0.73659 (15)	0.0384 (7)	
H7	0.3833	0.6674	0.7410	0.046*	
C10	0.29357 (7)	0.53409 (13)	0.29369 (11)	0.0148 (4)	
H10A	0.2720	0.4933	0.2756	0.018*	
H10B	0.3217	0.5242	0.2769	0.018*	
C11	0.32532 (7)	0.41959 (14)	0.39004 (11)	0.0171 (5)	
C12	0.34737 (8)	0.40255 (16)	0.44862 (12)	0.0235 (5)	
H12	0.3507	0.4433	0.4796	0.028*	
C13	0.36449 (9)	0.32471 (17)	0.46129 (14)	0.0311 (6)	
H13	0.3795	0.3128	0.5011	0.037*	
C14	0.35973 (9)	0.26519 (17)	0.41627 (16)	0.0340 (7)	
H14	0.3716	0.2127	0.4251	0.041*	
C15	0.33770 (9)	0.28230 (17)	0.35855 (16)	0.0345 (7)	
H15	0.3343	0.2413	0.3277	0.041*	
C16	0.32043 (8)	0.35929 (16)	0.34516 (14)	0.0263 (6)	
H16	0.3053	0.3706	0.3053	0.032*	
C20	0.43242 (7)	0.72999 (14)	0.34908 (11)	0.0152 (4)	
H20A	0.4608	0.7592	0.3567	0.018*	
H20B	0.4350	0.6894	0.3163	0.018*	
C21	0.24624 (7)	0.50760 (14)	0.39803 (11)	0.0147 (4)	
C22	0.21789 (8)	0.45073 (15)	0.36763 (12)	0.0227 (5)	
H22	0.2275	0.4183	0.3359	0.027*	
C23	0.17546 (8)	0.44093 (16)	0.38335 (13)	0.0262 (6)	
H23	0.1562	0.4021	0.3622	0.031*	
C24	0.16141 (8)	0.48786 (16)	0.42986 (12)	0.0215 (5)	
H24	0.1326	0.4807	0.4409	0.026*	
C25	0.18909 (8)	0.54496 (16)	0.46013 (12)	0.0234 (5)	
H25	0.1793	0.5774	0.4918	0.028*	
C26	0.23161 (8)	0.55497 (15)	0.44410 (11)	0.0199 (5)	
H26	0.2506	0.5944	0.4648	0.024*	
C31	0.21414 (7)	0.63403 (13)	0.27840 (11)	0.0153 (4)	
C32	0.19907 (7)	0.68226 (15)	0.32371 (12)	0.0197 (5)	
H32	0.2192	0.7149	0.3496	0.024*	
C33	0.15427 (8)	0.68285 (16)	0.33128 (13)	0.0258 (6)	
H33	0.1441	0.7156	0.3624	0.031*	
C34	0.12500 (8)	0.63577 (16)	0.29355 (13)	0.0242 (5)	
H34	0.0947	0.6360	0.2988	0.029*	
C35	0.13970 (8)	0.58808 (17)	0.24803 (13)	0.0261 (6)	
H35	0.1194	0.5563	0.2217	0.031*	
C36	0.18432 (8)	0.58659 (16)	0.24074 (12)	0.0224 (5)	
H36	0.1944	0.5531	0.2100	0.027*	
C41	0.27386 (7)	0.63926 (14)	0.18740 (11)	0.0160 (4)	
C42	0.26697 (8)	0.57103 (15)	0.14897 (12)	0.0204 (5)	
H42	0.2629	0.5193	0.1665	0.024*	
C43	0.26611 (8)	0.57888 (17)	0.08545 (12)	0.0261 (5)	
H43	0.2616	0.5324	0.0598	0.031*	
C44	0.27183 (9)	0.65415 (19)	0.05911 (12)	0.0287 (6)	
H44	0.2713	0.6591	0.0156	0.034*	
C45	0.27831 (9)	0.72187 (18)	0.09647 (13)	0.0285 (6)	
H45	0.2821	0.7735	0.0785	0.034*	
C46	0.27935 (8)	0.71476 (15)	0.16023 (12)	0.0213 (5)	
H46	0.2838	0.7616	0.1855	0.026*	
C51	0.46106 (7)	0.59204 (14)	0.42004 (11)	0.0150 (4)	
C52	0.44499 (7)	0.51591 (14)	0.43294 (11)	0.0179 (5)	
H52	0.4158	0.5109	0.4432	0.021*	
C53	0.47090 (8)	0.44711 (15)	0.43111 (12)	0.0216 (5)	
H53	0.4593	0.3953	0.4391	0.026*	
C54	0.51389 (8)	0.45446 (16)	0.41752 (12)	0.0238 (5)	
H54	0.5319	0.4077	0.4169	0.029*	
C55	0.53054 (8)	0.52986 (17)	0.40493 (12)	0.0242 (5)	
H55	0.5600	0.5347	0.3959	0.029*	
C56	0.50428 (8)	0.59852 (16)	0.40539 (11)	0.0208 (5)	
H56	0.5156	0.6499	0.3958	0.025*	
C61	0.44553 (7)	0.74433 (13)	0.48286 (10)	0.0142 (4)	
C62	0.49017 (8)	0.74738 (14)	0.50644 (12)	0.0200 (5)	
H62	0.5108	0.7143	0.4889	0.024*	
C63	0.50455 (8)	0.79865 (16)	0.55549 (13)	0.0275 (6)	
H63	0.5349	0.7999	0.5715	0.033*	
C64	0.47489 (9)	0.84788 (17)	0.58114 (13)	0.0272 (6)	
H64	0.4849	0.8830	0.6145	0.033*	
C65	0.43043 (9)	0.84565 (16)	0.55794 (12)	0.0245 (5)	
H65	0.4101	0.8797	0.5752	0.029*	
C66	0.41579 (8)	0.79381 (15)	0.50974 (11)	0.0193 (5)	
H66	0.3853	0.7917	0.4947	0.023*	
C71	0.40652 (7)	0.82620 (14)	0.24539 (11)	0.0162 (4)	
C72	0.39431 (8)	0.77072 (16)	0.19800 (12)	0.0214 (5)	
H72	0.3759	0.7263	0.2051	0.026*	
C73	0.40891 (9)	0.77995 (18)	0.14049 (13)	0.0275 (6)	
H73	0.4009	0.7413	0.1088	0.033*	
C74	0.43512 (8)	0.84561 (18)	0.12935 (12)	0.0275 (6)	
H74	0.4452	0.8519	0.0901	0.033*	
C75	0.44653 (8)	0.90190 (18)	0.17565 (13)	0.0271 (6)	
H75	0.4640	0.9474	0.1678	0.033*	
C76	0.43253 (8)	0.89235 (15)	0.23358 (12)	0.0207 (5)	
H76	0.4407	0.9310	0.2652	0.025*	
C81	0.40104 (7)	0.89352 (14)	0.36760 (11)	0.0159 (4)	
C82	0.36545 (8)	0.94055 (14)	0.38123 (11)	0.0185 (5)	
H82	0.3363	0.9250	0.3657	0.022*	
C83	0.37246 (9)	1.00972 (16)	0.41718 (12)	0.0252 (5)	
H83	0.3482	1.0419	0.4257	0.030*	
C84	0.41509 (9)	1.03167 (16)	0.44066 (13)	0.0274 (6)	
H84	0.4199	1.0788	0.4656	0.033*	
C85	0.45069 (9)	0.98535 (17)	0.42795 (13)	0.0274 (6)	
H85	0.4797	1.0007	0.4442	0.033*	
C86	0.44392 (8)	0.91653 (15)	0.39146 (12)	0.0209 (5)	
H86	0.4683	0.8850	0.3826	0.025*	
N3	0.5000	0.0799 (3)	0.2500	0.0509 (11)	
C8	0.5000	0.1474 (3)	0.2500	0.0472 (12)	
C9	0.5000	0.2356 (4)	0.2500	0.114 (3)	
H9A	0.5194	0.2550	0.2219	0.171*	0.5
H9B	0.4707	0.2550	0.2372	0.171*	0.5
H9C	0.5099	0.2550	0.2909	0.171*	0.5

### Atomic displacement parameters (Å^2^)

**Table:**

	*U*^11^	*U*^22^	*U*^33^	*U*^12^	*U*^13^	*U*^23^
Ag1	0.01265 (8)	0.01232 (8)	0.01462 (9)	0.00062 (6)	0.00176 (6)	−0.00100 (6)
Ag2	0.01315 (8)	0.01121 (8)	0.01675 (9)	−0.00039 (6)	0.00076 (6)	−0.00092 (6)
Cl1	0.0165 (2)	0.0153 (3)	0.0160 (3)	0.00499 (19)	0.00185 (19)	−0.0005 (2)
Cl2	0.0170 (2)	0.0160 (3)	0.0208 (3)	0.00423 (19)	0.0038 (2)	0.0054 (2)
S1	0.0151 (2)	0.0261 (3)	0.0135 (3)	0.0066 (2)	0.0036 (2)	0.0031 (2)
P1	0.0133 (2)	0.0107 (3)	0.0145 (3)	0.00021 (19)	0.0021 (2)	0.0001 (2)
P2	0.0136 (2)	0.0111 (3)	0.0129 (3)	−0.00047 (19)	0.0012 (2)	−0.0005 (2)
P3	0.0117 (2)	0.0126 (3)	0.0122 (3)	0.00089 (19)	0.0016 (2)	0.0008 (2)
P4	0.0129 (2)	0.0123 (3)	0.0129 (3)	−0.0004 (2)	0.0013 (2)	0.0006 (2)
N1	0.0171 (9)	0.0257 (11)	0.0163 (11)	0.0029 (8)	0.0056 (8)	0.0006 (8)
N2	0.0147 (9)	0.0251 (11)	0.0149 (10)	0.0049 (8)	0.0003 (7)	−0.0039 (8)
C1	0.0139 (10)	0.0151 (11)	0.0153 (12)	−0.0016 (8)	0.0030 (8)	−0.0034 (9)
C2	0.0212 (11)	0.0290 (14)	0.0160 (12)	−0.0015 (10)	0.0060 (9)	0.0058 (10)
C3	0.0201 (11)	0.0350 (15)	0.0178 (13)	0.0031 (10)	0.0067 (9)	0.0040 (11)
C4	0.0264 (13)	0.0379 (16)	0.0246 (15)	0.0067 (11)	0.0093 (11)	0.0134 (12)
C5	0.0336 (15)	0.052 (2)	0.0297 (17)	−0.0015 (14)	−0.0076 (12)	0.0210 (14)
C6	0.064 (2)	0.043 (2)	0.036 (2)	−0.0120 (17)	−0.0238 (16)	0.0102 (15)
C7	0.0529 (18)	0.0304 (16)	0.0282 (17)	−0.0040 (13)	−0.0099 (13)	0.0099 (13)
C10	0.0164 (10)	0.0123 (10)	0.0163 (12)	0.0002 (8)	0.0044 (8)	−0.0011 (8)
C11	0.0149 (10)	0.0137 (11)	0.0229 (13)	−0.0004 (8)	0.0032 (9)	0.0018 (9)
C12	0.0296 (13)	0.0195 (12)	0.0216 (14)	0.0000 (10)	0.0043 (10)	0.0020 (10)
C13	0.0351 (14)	0.0257 (14)	0.0316 (16)	0.0057 (11)	0.0002 (12)	0.0109 (12)
C14	0.0328 (14)	0.0162 (13)	0.051 (2)	0.0078 (11)	−0.0003 (13)	0.0064 (12)
C15	0.0342 (14)	0.0180 (13)	0.049 (2)	0.0058 (11)	−0.0034 (13)	−0.0067 (13)
C16	0.0253 (12)	0.0185 (13)	0.0326 (16)	0.0055 (10)	−0.0061 (11)	−0.0046 (11)
C20	0.0132 (10)	0.0164 (11)	0.0166 (12)	0.0016 (8)	0.0041 (8)	0.0039 (9)
C21	0.0163 (10)	0.0137 (11)	0.0143 (11)	0.0004 (8)	0.0029 (8)	0.0019 (9)
C22	0.0216 (11)	0.0223 (13)	0.0258 (14)	−0.0041 (9)	0.0088 (10)	−0.0110 (10)
C23	0.0209 (12)	0.0262 (14)	0.0323 (15)	−0.0090 (10)	0.0064 (10)	−0.0095 (11)
C24	0.0166 (11)	0.0256 (13)	0.0232 (13)	−0.0015 (9)	0.0062 (9)	0.0005 (10)
C25	0.0248 (12)	0.0246 (13)	0.0229 (13)	−0.0020 (10)	0.0110 (10)	−0.0059 (10)
C26	0.0217 (11)	0.0197 (12)	0.0188 (13)	−0.0060 (9)	0.0046 (9)	−0.0048 (9)
C31	0.0163 (10)	0.0114 (10)	0.0181 (12)	0.0012 (8)	0.0020 (8)	0.0033 (9)
C32	0.0180 (11)	0.0188 (12)	0.0227 (13)	−0.0022 (9)	0.0038 (9)	−0.0027 (10)
C33	0.0236 (12)	0.0262 (14)	0.0298 (15)	−0.0012 (10)	0.0114 (11)	−0.0055 (11)
C34	0.0169 (11)	0.0251 (13)	0.0315 (15)	−0.0006 (9)	0.0064 (10)	0.0031 (11)
C35	0.0205 (12)	0.0279 (14)	0.0295 (15)	−0.0051 (10)	0.0021 (10)	−0.0039 (11)
C36	0.0174 (11)	0.0242 (13)	0.0256 (14)	−0.0027 (9)	0.0028 (9)	−0.0074 (10)
C41	0.0137 (10)	0.0172 (11)	0.0172 (12)	−0.0015 (8)	0.0024 (8)	−0.0015 (9)
C42	0.0222 (11)	0.0197 (12)	0.0194 (13)	−0.0019 (9)	0.0037 (9)	−0.0020 (10)
C43	0.0282 (13)	0.0307 (14)	0.0187 (13)	0.0011 (11)	0.0004 (10)	−0.0051 (11)
C44	0.0314 (13)	0.0416 (17)	0.0129 (13)	0.0030 (12)	0.0024 (10)	0.0025 (11)
C45	0.0357 (14)	0.0289 (14)	0.0207 (14)	−0.0009 (11)	0.0030 (11)	0.0080 (11)
C46	0.0249 (12)	0.0187 (12)	0.0202 (13)	−0.0017 (9)	0.0021 (10)	0.0021 (10)
C51	0.0138 (10)	0.0183 (11)	0.0127 (11)	0.0057 (8)	0.0006 (8)	−0.0001 (9)
C52	0.0185 (10)	0.0172 (11)	0.0178 (12)	0.0034 (9)	0.0023 (9)	0.0006 (9)
C53	0.0268 (12)	0.0168 (12)	0.0202 (13)	0.0054 (9)	−0.0008 (10)	−0.0008 (9)
C54	0.0242 (12)	0.0278 (14)	0.0184 (13)	0.0140 (10)	−0.0010 (10)	−0.0030 (10)
C55	0.0176 (11)	0.0378 (15)	0.0174 (13)	0.0099 (10)	0.0031 (9)	0.0018 (11)
C56	0.0193 (11)	0.0271 (13)	0.0161 (12)	0.0025 (9)	0.0031 (9)	0.0050 (10)
C61	0.0161 (10)	0.0145 (11)	0.0118 (11)	−0.0022 (8)	0.0015 (8)	0.0015 (8)
C62	0.0175 (11)	0.0170 (12)	0.0245 (13)	−0.0013 (9)	−0.0014 (9)	0.0023 (10)
C63	0.0224 (12)	0.0257 (14)	0.0318 (16)	−0.0061 (10)	−0.0066 (10)	−0.0001 (11)
C64	0.0350 (14)	0.0246 (13)	0.0206 (14)	−0.0127 (11)	−0.0019 (11)	−0.0042 (11)
C65	0.0300 (13)	0.0234 (13)	0.0209 (13)	−0.0047 (10)	0.0067 (10)	−0.0033 (10)
C66	0.0173 (10)	0.0222 (12)	0.0185 (12)	−0.0026 (9)	0.0028 (9)	0.0001 (10)
C71	0.0153 (10)	0.0162 (11)	0.0171 (12)	0.0033 (8)	0.0021 (8)	0.0040 (9)
C72	0.0227 (11)	0.0227 (12)	0.0181 (13)	−0.0001 (9)	0.0001 (9)	0.0011 (10)
C73	0.0270 (13)	0.0358 (15)	0.0193 (14)	0.0069 (11)	0.0012 (10)	−0.0039 (11)
C74	0.0199 (12)	0.0449 (17)	0.0190 (13)	0.0063 (11)	0.0072 (10)	0.0066 (12)
C75	0.0224 (12)	0.0342 (15)	0.0261 (15)	0.0003 (10)	0.0082 (10)	0.0078 (11)
C76	0.0200 (11)	0.0205 (12)	0.0217 (13)	0.0018 (9)	0.0029 (9)	0.0040 (10)
C81	0.0200 (10)	0.0149 (11)	0.0129 (11)	−0.0014 (8)	0.0027 (8)	0.0011 (9)
C82	0.0207 (11)	0.0169 (11)	0.0183 (12)	−0.0042 (9)	0.0038 (9)	−0.0007 (9)
C83	0.0333 (13)	0.0189 (12)	0.0247 (14)	−0.0007 (10)	0.0093 (11)	−0.0020 (10)
C84	0.0428 (15)	0.0181 (12)	0.0214 (14)	−0.0099 (11)	0.0040 (11)	−0.0061 (10)
C85	0.0324 (13)	0.0255 (14)	0.0228 (14)	−0.0134 (11)	−0.0028 (11)	0.0016 (11)
C86	0.0189 (11)	0.0199 (12)	0.0238 (13)	−0.0047 (9)	0.0021 (9)	0.0025 (10)
N3	0.051 (2)	0.047 (3)	0.051 (3)	0.000	−0.010 (2)	0.000
C8	0.042 (3)	0.052 (3)	0.043 (3)	0.000	−0.011 (2)	0.000
C9	0.165 (9)	0.043 (4)	0.120 (8)	0.000	−0.036 (6)	0.000

### Geometric parameters (Å, º)

**Table:**

Ag1—P1	2.4548 (6)	C32—H32	0.9500
Ag1—P3	2.5060 (6)	C33—C34	1.379 (4)
Ag1—Cl1	2.6422 (5)	C33—H33	0.9500
Ag1—S1	2.6445 (6)	C34—C35	1.385 (4)
Ag1—Ag2	3.2064 (2)	C34—H34	0.9500
Ag2—P2	2.4403 (6)	C35—C36	1.395 (3)
Ag2—P4	2.4555 (6)	C35—H35	0.9500
Ag2—Cl2	2.6470 (6)	C36—H36	0.9500
Ag2—Cl1	2.6933 (6)	C41—C46	1.399 (3)
S1—C1	1.708 (2)	C41—C42	1.404 (3)
P1—C21	1.832 (2)	C42—C43	1.388 (4)
P1—C11	1.836 (2)	C42—H42	0.9500
P1—C10	1.843 (2)	C43—C44	1.389 (4)
P2—C41	1.822 (2)	C43—H43	0.9500
P2—C31	1.839 (2)	C44—C45	1.383 (4)
P2—C10	1.845 (2)	C44—H44	0.9500
P3—C51	1.826 (2)	C45—C46	1.391 (4)
P3—C61	1.828 (2)	C45—H45	0.9500
P3—C20	1.848 (2)	C46—H46	0.9500
P4—C81	1.820 (2)	C51—C52	1.391 (3)
P4—C71	1.838 (2)	C51—C56	1.403 (3)
P4—C20	1.846 (2)	C52—C53	1.389 (3)
N1—C1	1.342 (3)	C52—H52	0.9500
N1—C2	1.429 (3)	C53—C54	1.390 (3)
N1—H1	0.8800	C53—H53	0.9500
N2—C1	1.331 (3)	C54—C55	1.385 (4)
N2—H2A	0.8800	C54—H54	0.9500
N2—H2B	0.8800	C55—C56	1.390 (3)
C2—C7	1.379 (4)	C55—H55	0.9500
C2—C3	1.383 (4)	C56—H56	0.9500
C3—C4	1.399 (4)	C61—C62	1.400 (3)
C3—H3	0.9500	C61—C66	1.404 (3)
C4—C5	1.385 (5)	C62—C63	1.392 (4)
C4—H4	0.9500	C62—H62	0.9500
C5—C6	1.372 (5)	C63—C64	1.387 (4)
C5—H5	0.9500	C63—H63	0.9500
C6—C7	1.395 (4)	C64—C65	1.392 (4)
C6—H6	0.9500	C64—H64	0.9500
C7—H7	0.9500	C65—C66	1.386 (4)
C10—H10A	0.9900	C65—H65	0.9500
C10—H10B	0.9900	C66—H66	0.9500
C11—C16	1.391 (4)	C71—C76	1.394 (3)
C11—C12	1.397 (4)	C71—C72	1.396 (3)
C12—C13	1.402 (4)	C72—C73	1.390 (4)
C12—H12	0.9500	C72—H72	0.9500
C13—C14	1.383 (4)	C73—C74	1.388 (4)
C13—H13	0.9500	C73—H73	0.9500
C14—C15	1.381 (4)	C74—C75	1.384 (4)
C14—H14	0.9500	C74—H74	0.9500
C15—C16	1.393 (4)	C75—C76	1.392 (3)
C15—H15	0.9500	C75—H75	0.9500
C16—H16	0.9500	C76—H76	0.9500
C20—H20A	0.9900	C81—C82	1.399 (3)
C20—H20B	0.9900	C81—C86	1.403 (3)
C21—C22	1.390 (3)	C82—C83	1.386 (3)
C21—C26	1.390 (3)	C82—H82	0.9500
C22—C23	1.393 (3)	C83—C84	1.390 (4)
C22—H22	0.9500	C83—H83	0.9500
C23—C24	1.386 (3)	C84—C85	1.387 (4)
C23—H23	0.9500	C84—H84	0.9500
C24—C25	1.380 (4)	C85—C86	1.387 (4)
C24—H24	0.9500	C85—H85	0.9500
C25—C26	1.399 (3)	C86—H86	0.9500
C25—H25	0.9500	N3—C8	1.113 (7)
C26—H26	0.9500	C8—C9	1.456 (9)
C31—C36	1.391 (3)	C9—H9A	0.9600
C31—C32	1.391 (3)	C9—H9B	0.9600
C32—C33	1.402 (3)	C9—H9C	0.9600
			
P1—Ag1—P3	129.466 (19)	C21—C26—C25	120.5 (2)
P1—Ag1—Cl1	110.406 (19)	C21—C26—H26	119.8
P3—Ag1—Cl1	104.346 (18)	C25—C26—H26	119.8
P1—Ag1—S1	115.43 (2)	C36—C31—C32	119.3 (2)
P3—Ag1—S1	92.061 (19)	C36—C31—P2	122.72 (17)
Cl1—Ag1—S1	100.632 (18)	C32—C31—P2	117.95 (18)
P1—Ag1—Ag2	87.379 (15)	C31—C32—C33	120.2 (2)
P3—Ag1—Ag2	84.618 (14)	C31—C32—H32	119.9
Cl1—Ag1—Ag2	53.794 (13)	C33—C32—H32	119.9
S1—Ag1—Ag2	151.860 (15)	C34—C33—C32	119.9 (2)
P2—Ag2—P4	134.00 (2)	C34—C33—H33	120.0
P2—Ag2—Cl2	100.775 (19)	C32—C33—H33	120.0
P4—Ag2—Cl2	105.042 (19)	C33—C34—C35	120.2 (2)
P2—Ag2—Cl1	112.368 (19)	C33—C34—H34	119.9
P4—Ag2—Cl1	104.589 (19)	C35—C34—H34	119.9
Cl2—Ag2—Cl1	91.050 (17)	C34—C35—C36	120.1 (2)
P2—Ag2—Ag1	89.045 (15)	C34—C35—H35	119.9
P4—Ag2—Ag1	92.677 (15)	C36—C35—H35	119.9
Cl2—Ag2—Ag1	142.687 (14)	C31—C36—C35	120.2 (2)
Cl1—Ag2—Ag1	52.334 (12)	C31—C36—H36	119.9
Ag1—Cl1—Ag2	73.872 (14)	C35—C36—H36	119.9
C1—S1—Ag1	109.82 (8)	C46—C41—C42	118.6 (2)
C21—P1—C11	101.82 (10)	C46—C41—P2	117.58 (18)
C21—P1—C10	103.09 (10)	C42—C41—P2	123.71 (18)
C11—P1—C10	104.14 (11)	C43—C42—C41	120.2 (2)
C21—P1—Ag1	117.13 (8)	C43—C42—H42	119.9
C11—P1—Ag1	113.41 (8)	C41—C42—H42	119.9
C10—P1—Ag1	115.47 (7)	C42—C43—C44	120.5 (3)
C41—P2—C31	102.79 (10)	C42—C43—H43	119.7
C41—P2—C10	104.42 (10)	C44—C43—H43	119.7
C31—P2—C10	105.02 (10)	C45—C44—C43	119.7 (2)
C41—P2—Ag2	112.53 (7)	C45—C44—H44	120.1
C31—P2—Ag2	115.55 (8)	C43—C44—H44	120.1
C10—P2—Ag2	115.17 (8)	C44—C45—C46	120.3 (3)
C51—P3—C61	106.56 (10)	C44—C45—H45	119.9
C51—P3—C20	100.74 (10)	C46—C45—H45	119.9
C61—P3—C20	104.31 (10)	C45—C46—C41	120.6 (2)
C51—P3—Ag1	110.80 (8)	C45—C46—H46	119.7
C61—P3—Ag1	112.33 (7)	C41—C46—H46	119.7
C20—P3—Ag1	120.72 (7)	C52—C51—C56	118.8 (2)
C81—P4—C71	105.44 (11)	C52—C51—P3	116.41 (16)
C81—P4—C20	105.12 (11)	C56—C51—P3	124.72 (19)
C71—P4—C20	98.72 (10)	C53—C52—C51	121.0 (2)
C81—P4—Ag2	114.69 (7)	C53—C52—H52	119.5
C71—P4—Ag2	118.44 (8)	C51—C52—H52	119.5
C20—P4—Ag2	112.53 (7)	C52—C53—C54	119.6 (2)
C1—N1—C2	125.16 (19)	C52—C53—H53	120.2
C1—N1—H1	117.4	C54—C53—H53	120.2
C2—N1—H1	117.4	C55—C54—C53	120.1 (2)
C1—N2—H2A	120.0	C55—C54—H54	119.9
C1—N2—H2B	120.0	C53—C54—H54	119.9
H2A—N2—H2B	120.0	C54—C55—C56	120.2 (2)
N2—C1—N1	116.19 (19)	C54—C55—H55	119.9
N2—C1—S1	122.04 (18)	C56—C55—H55	119.9
N1—C1—S1	121.77 (17)	C55—C56—C51	120.1 (2)
C7—C2—C3	120.3 (3)	C55—C56—H56	119.9
C7—C2—N1	118.5 (2)	C51—C56—H56	119.9
C3—C2—N1	121.1 (2)	C62—C61—C66	118.7 (2)
C2—C3—C4	119.4 (3)	C62—C61—P3	124.12 (18)
C2—C3—H3	120.3	C66—C61—P3	117.16 (17)
C4—C3—H3	120.3	C63—C62—C61	120.4 (2)
C5—C4—C3	120.2 (3)	C63—C62—H62	119.8
C5—C4—H4	119.9	C61—C62—H62	119.8
C3—C4—H4	119.9	C64—C63—C62	120.4 (2)
C6—C5—C4	119.8 (3)	C64—C63—H63	119.8
C6—C5—H5	120.1	C62—C63—H63	119.8
C4—C5—H5	120.1	C63—C64—C65	119.8 (2)
C5—C6—C7	120.3 (3)	C63—C64—H64	120.1
C5—C6—H6	119.8	C65—C64—H64	120.1
C7—C6—H6	119.8	C66—C65—C64	120.1 (2)
C2—C7—C6	119.9 (3)	C66—C65—H65	120.0
C2—C7—H7	120.0	C64—C65—H65	120.0
C6—C7—H7	120.0	C65—C66—C61	120.7 (2)
P1—C10—P2	111.83 (11)	C65—C66—H66	119.7
P1—C10—H10A	109.3	C61—C66—H66	119.7
P2—C10—H10A	109.3	C76—C71—C72	118.9 (2)
P1—C10—H10B	109.3	C76—C71—P4	124.59 (19)
P2—C10—H10B	109.3	C72—C71—P4	116.42 (18)
H10A—C10—H10B	107.9	C73—C72—C71	120.6 (2)
C16—C11—C12	119.8 (2)	C73—C72—H72	119.7
C16—C11—P1	123.70 (19)	C71—C72—H72	119.7
C12—C11—P1	116.48 (18)	C74—C73—C72	120.1 (3)
C11—C12—C13	119.4 (3)	C74—C73—H73	120.0
C11—C12—H12	120.3	C72—C73—H73	120.0
C13—C12—H12	120.3	C75—C74—C73	119.7 (2)
C14—C13—C12	120.5 (3)	C75—C74—H74	120.1
C14—C13—H13	119.8	C73—C74—H74	120.1
C12—C13—H13	119.8	C74—C75—C76	120.5 (2)
C15—C14—C13	119.8 (3)	C74—C75—H75	119.8
C15—C14—H14	120.1	C76—C75—H75	119.8
C13—C14—H14	120.1	C75—C76—C71	120.2 (2)
C14—C15—C16	120.5 (3)	C75—C76—H76	119.9
C14—C15—H15	119.8	C71—C76—H76	119.9
C16—C15—H15	119.8	C82—C81—C86	119.2 (2)
C11—C16—C15	120.1 (3)	C82—C81—P4	117.55 (17)
C11—C16—H16	120.0	C86—C81—P4	123.20 (18)
C15—C16—H16	120.0	C83—C82—C81	120.5 (2)
P4—C20—P3	113.90 (11)	C83—C82—H82	119.8
P4—C20—H20A	108.8	C81—C82—H82	119.8
P3—C20—H20A	108.8	C82—C83—C84	119.7 (2)
P4—C20—H20B	108.8	C82—C83—H83	120.2
P3—C20—H20B	108.8	C84—C83—H83	120.2
H20A—C20—H20B	107.7	C85—C84—C83	120.5 (2)
C22—C21—C26	119.1 (2)	C85—C84—H84	119.7
C22—C21—P1	121.47 (17)	C83—C84—H84	119.7
C26—C21—P1	119.42 (17)	C84—C85—C86	120.0 (2)
C21—C22—C23	120.5 (2)	C84—C85—H85	120.0
C21—C22—H22	119.8	C86—C85—H85	120.0
C23—C22—H22	119.8	C85—C86—C81	120.0 (2)
C24—C23—C22	119.9 (2)	C85—C86—H86	120.0
C24—C23—H23	120.1	C81—C86—H86	120.0
C22—C23—H23	120.1	N3—C8—C9	180.0
C25—C24—C23	120.2 (2)	C8—C9—H9A	109.5
C25—C24—H24	119.9	C8—C9—H9B	109.5
C23—C24—H24	119.9	H9A—C9—H9B	109.5
C24—C25—C26	119.8 (2)	C8—C9—H9C	109.5
C24—C25—H25	120.1	H9A—C9—H9C	109.5
C26—C25—H25	120.1	H9B—C9—H9C	109.5
			
C2—N1—C1—N2	−176.2 (2)	C10—P2—C41—C46	−152.26 (18)
C2—N1—C1—S1	3.6 (3)	Ag2—P2—C41—C46	−26.7 (2)
Ag1—S1—C1—N2	−7.5 (2)	C31—P2—C41—C42	−78.4 (2)
Ag1—S1—C1—N1	172.72 (17)	C10—P2—C41—C42	31.0 (2)
C1—N1—C2—C7	109.2 (3)	Ag2—P2—C41—C42	156.62 (17)
C1—N1—C2—C3	−73.7 (3)	C46—C41—C42—C43	0.6 (3)
C7—C2—C3—C4	−1.1 (4)	P2—C41—C42—C43	177.34 (18)
N1—C2—C3—C4	−178.1 (2)	C41—C42—C43—C44	−0.3 (4)
C2—C3—C4—C5	0.6 (4)	C42—C43—C44—C45	−0.2 (4)
C3—C4—C5—C6	0.6 (4)	C43—C44—C45—C46	0.3 (4)
C4—C5—C6—C7	−1.3 (5)	C44—C45—C46—C41	0.0 (4)
C3—C2—C7—C6	0.5 (4)	C42—C41—C46—C45	−0.5 (4)
N1—C2—C7—C6	177.6 (3)	P2—C41—C46—C45	−177.4 (2)
C5—C6—C7—C2	0.8 (5)	C61—P3—C51—C52	116.89 (19)
C21—P1—C10—P2	77.44 (13)	C20—P3—C51—C52	−134.51 (19)
C11—P1—C10—P2	−176.57 (11)	Ag1—P3—C51—C52	−5.6 (2)
Ag1—P1—C10—P2	−51.55 (13)	C61—P3—C51—C56	−65.6 (2)
C41—P2—C10—P1	172.99 (11)	C20—P3—C51—C56	43.0 (2)
C31—P2—C10—P1	−79.21 (14)	Ag1—P3—C51—C56	171.93 (19)
Ag2—P2—C10—P1	49.07 (12)	C56—C51—C52—C53	−0.4 (4)
C21—P1—C11—C16	85.6 (2)	P3—C51—C52—C53	177.28 (19)
C10—P1—C11—C16	−21.4 (2)	C51—C52—C53—C54	1.4 (4)
Ag1—P1—C11—C16	−147.70 (19)	C52—C53—C54—C55	−1.0 (4)
C21—P1—C11—C12	−91.87 (19)	C53—C54—C55—C56	−0.4 (4)
C10—P1—C11—C12	161.19 (18)	C54—C55—C56—C51	1.5 (4)
Ag1—P1—C11—C12	34.9 (2)	C52—C51—C56—C55	−1.1 (4)
C16—C11—C12—C13	0.3 (4)	P3—C51—C56—C55	−178.53 (19)
P1—C11—C12—C13	177.82 (19)	C51—P3—C61—C62	23.1 (2)
C11—C12—C13—C14	0.1 (4)	C20—P3—C61—C62	−82.9 (2)
C12—C13—C14—C15	−0.4 (4)	Ag1—P3—C61—C62	144.64 (18)
C13—C14—C15—C16	0.4 (5)	C51—P3—C61—C66	−154.37 (18)
C12—C11—C16—C15	−0.4 (4)	C20—P3—C61—C66	99.57 (19)
P1—C11—C16—C15	−177.7 (2)	Ag1—P3—C61—C66	−32.9 (2)
C14—C15—C16—C11	0.0 (4)	C66—C61—C62—C63	−0.1 (4)
C81—P4—C20—P3	80.76 (14)	P3—C61—C62—C63	−177.56 (19)
C71—P4—C20—P3	−170.55 (13)	C61—C62—C63—C64	−0.6 (4)
Ag2—P4—C20—P3	−44.73 (14)	C62—C63—C64—C65	0.4 (4)
C51—P3—C20—P4	163.44 (13)	C63—C64—C65—C66	0.6 (4)
C61—P3—C20—P4	−86.20 (14)	C64—C65—C66—C61	−1.4 (4)
Ag1—P3—C20—P4	41.22 (16)	C62—C61—C66—C65	1.1 (3)
C11—P1—C21—C22	−57.0 (2)	P3—C61—C66—C65	178.75 (19)
C10—P1—C21—C22	50.8 (2)	C81—P4—C71—C76	12.8 (2)
Ag1—P1—C21—C22	178.74 (18)	C20—P4—C71—C76	−95.7 (2)
C11—P1—C21—C26	123.4 (2)	Ag2—P4—C71—C76	142.73 (18)
C10—P1—C21—C26	−128.8 (2)	C81—P4—C71—C72	−170.29 (18)
Ag1—P1—C21—C26	−0.8 (2)	C20—P4—C71—C72	81.27 (19)
C26—C21—C22—C23	−0.4 (4)	Ag2—P4—C71—C72	−40.3 (2)
P1—C21—C22—C23	−180.0 (2)	C76—C71—C72—C73	1.9 (4)
C21—C22—C23—C24	−0.4 (4)	P4—C71—C72—C73	−175.26 (19)
C22—C23—C24—C25	0.8 (4)	C71—C72—C73—C74	−1.3 (4)
C23—C24—C25—C26	−0.5 (4)	C72—C73—C74—C75	−0.2 (4)
C22—C21—C26—C25	0.7 (4)	C73—C74—C75—C76	1.1 (4)
P1—C21—C26—C25	−179.7 (2)	C74—C75—C76—C71	−0.5 (4)
C24—C25—C26—C21	−0.2 (4)	C72—C71—C76—C75	−0.9 (4)
C41—P2—C31—C36	42.3 (2)	P4—C71—C76—C75	175.93 (19)
C10—P2—C31—C36	−66.7 (2)	C71—P4—C81—C82	108.82 (19)
Ag2—P2—C31—C36	165.25 (18)	C20—P4—C81—C82	−147.44 (18)
C41—P2—C31—C32	−137.48 (19)	Ag2—P4—C81—C82	−23.3 (2)
C10—P2—C31—C32	113.5 (2)	C71—P4—C81—C86	−73.0 (2)
Ag2—P2—C31—C32	−14.5 (2)	C20—P4—C81—C86	30.8 (2)
C36—C31—C32—C33	0.1 (4)	Ag2—P4—C81—C86	154.91 (18)
P2—C31—C32—C33	179.9 (2)	C86—C81—C82—C83	0.9 (4)
C31—C32—C33—C34	−0.3 (4)	P4—C81—C82—C83	179.17 (19)
C32—C33—C34—C35	−0.2 (4)	C81—C82—C83—C84	−1.1 (4)
C33—C34—C35—C36	0.9 (4)	C82—C83—C84—C85	0.6 (4)
C32—C31—C36—C35	0.6 (4)	C83—C84—C85—C86	0.1 (4)
P2—C31—C36—C35	−179.2 (2)	C84—C85—C86—C81	−0.3 (4)
C34—C35—C36—C31	−1.1 (4)	C82—C81—C86—C85	−0.2 (4)
C31—P2—C41—C46	98.31 (19)	P4—C81—C86—C85	−178.40 (19)

### Hydrogen-bond geometry (Å, º)

**Table:**

*D*—H···*A*	*D*—H	H···*A*	*D*···*A*	*D*—H···*A*
N1—H1···Cl2^i^	0.88	2.32	3.1910 (19)	172
N2—H2*A*···Cl1^i^	0.88	2.56	3.1618 (19)	126
N2—H2*A*···Cl2^i^	0.88	2.84	3.595 (2)	144
N2—H2*B*···Cl1	0.88	2.52	3.328 (2)	152

Symmetry code: (i) −*x*+1/2, −*y*+3/2, −*z*+1.
