# Supplementary figures and images for: Crystal structure of bis­[μ-bis­(di­phenyl­phosphan­yl)methane-κ2 P:P′]-μ-chlorido-chlorido-1κCl-(1-phenyl­thio­urea-2κS)disilver aceto­nitrile hemisolvate
Source: Acta Crystallogr E Crystallogr Commun. 2015 May 23;71(Pt 6):m133–4. doi: 10.1107/S2056989015008981 (PMC4459320; doi:10.1107/S2056989015008981)

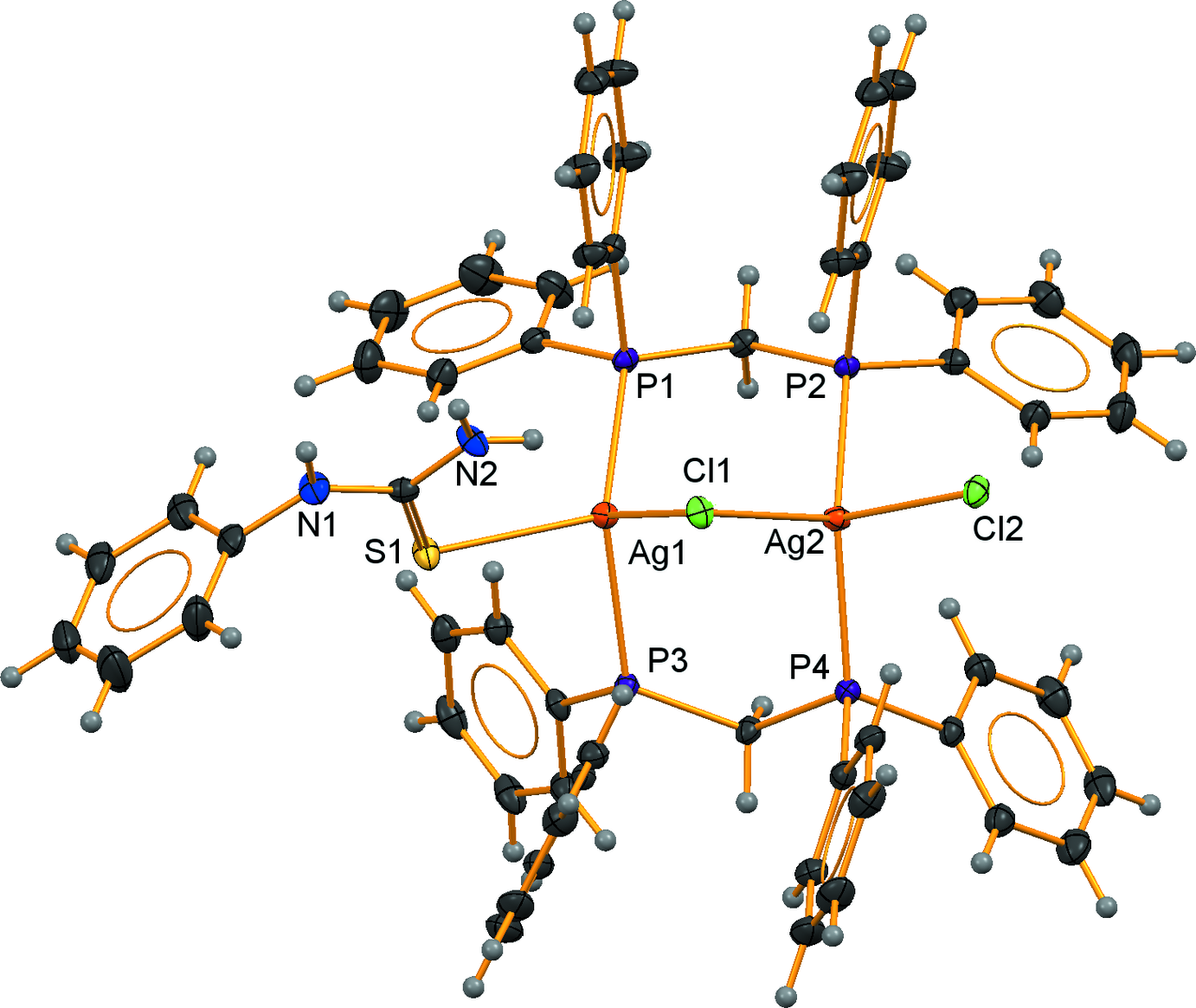

Supplement: Supplementary file 3 [file e-71-0m133-fig1.tif]

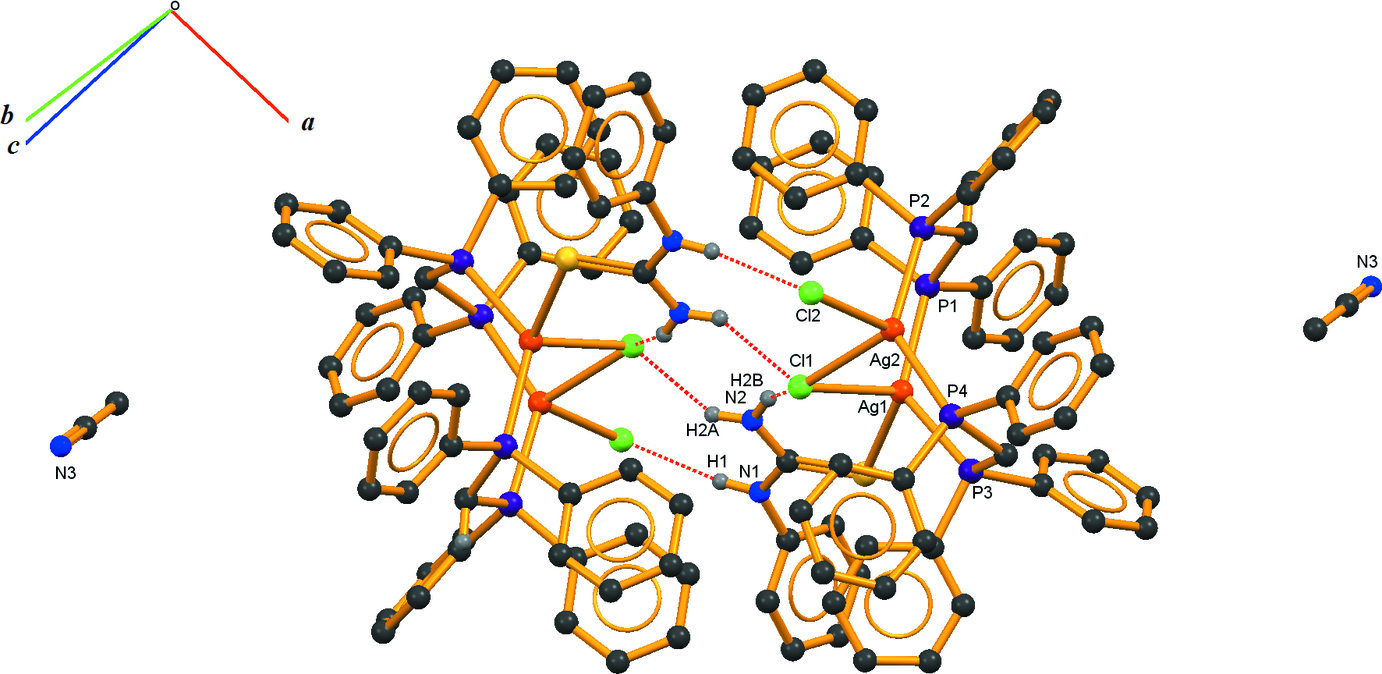

Supplement: Supplementary file 4 [file e-71-0m133-fig2.tif]

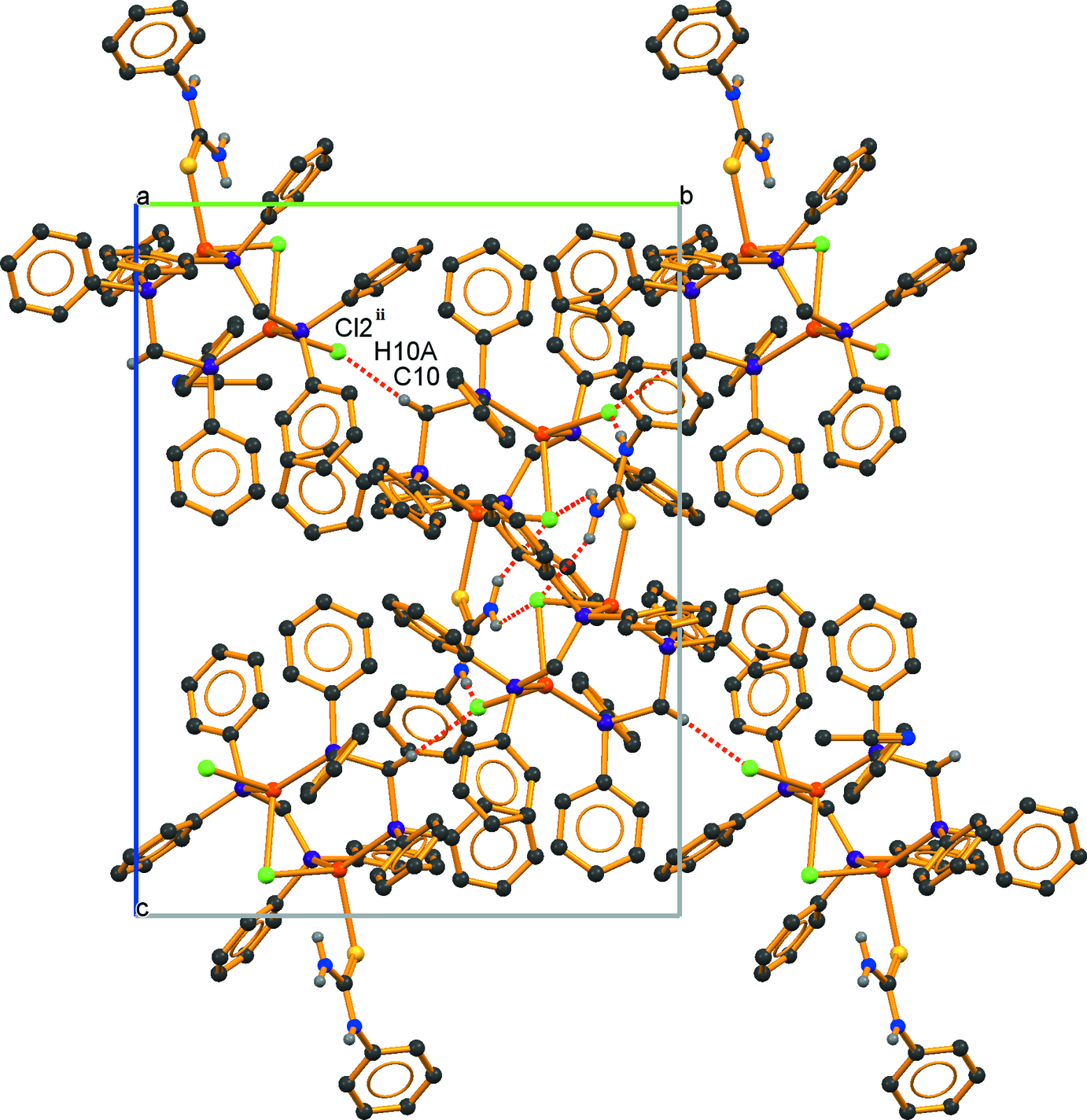

Supplement: Supplementary file 5 [file e-71-0m133-fig3.tif]
